# Contact with Domestic Dogs Increases Pathogen Exposure in Endangered African Wild Dogs (*Lycaon pictus*)

**DOI:** 10.1371/journal.pone.0030099

**Published:** 2012-01-06

**Authors:** Rosie Woodroffe, Katherine C. Prager, Linda Munson, Patricia A. Conrad, Edward J. Dubovi, Jonna A. K. Mazet

**Affiliations:** 1 Institute of Zoology, Regent's Park, London, United Kingdom; 2 Wildlife Health Center, University of California Davis, Davis, California, United States of America; 3 Department of Pathology, Microbiology and Immunology, University of California Davis, Davis, California, United States of America; 4 Animal Health Diagnostic Center, College of Veterinary Medicine, Cornell University, Ithaca, New York, United States of America; University of California, Berkeley, United States of America

## Abstract

**Background:**

Infectious diseases have contributed to the decline and local extinction of several wildlife species, including African wild dogs (*Lycaon pictus*). Mitigating such disease threats is challenging, partly because uncertainty about disease dynamics makes it difficult to identify the best management approaches. Serious impacts on susceptible populations most frequently occur when generalist pathogens are maintained within populations of abundant (often domestic) “reservoir” hosts, and spill over into less abundant host species. If this is the case, disease control directed at the reservoir host might be most appropriate. However, pathogen transmission within threatened host populations may also be important, and may not be controllable by managing another host species.

**Methodology/Principal Findings:**

We investigated interspecific and intraspecific transmission routes, by comparing African wild dogs' exposure to six canine pathogens with behavioural measures of their opportunities for contact with domestic dogs and with other wild dogs. Domestic dog contact was associated with exposure to canine parvovirus, *Ehrlichia canis*, *Neospora caninum* and perhaps rabies virus, but not with exposure to canine distemper virus or canine coronavirus. Contact with other wild dogs appeared not to increase the risk of exposure to any of the pathogens.

**Conclusions/Significance:**

These findings, combined with other data, suggest that management directed at domestic dogs might help to protect wild dog populations from rabies virus, but not from canine distemper virus. However, further analyses are needed to determine the management approaches – including no intervention – which are most appropriate for each pathogen.

## Introduction

Infectious diseases have contributed to the decline and local extinction of several wildlife species [1], [2], [3], and may threaten many more. However, mitigation of disease threats is challenging, partly because managers often lack the information needed to select the most appropriate management approaches.

Serious impacts on host populations most frequently occur when generalist pathogens are maintained within populations of abundant “reservoir” hosts and “spill-over” into less abundant, and potentially more susceptible, host species [4], [5]. Such situations represent a form of parasite-mediated apparent competition [6] which can threaten the persistence of spill-over host species [1]. In principle, such disease threats could be mitigated by management (e.g. vaccination) directed at reservoir hosts. This approach may be attractive, especially where – as is often the case [7] – reservoir hosts are domestic animals which can be managed more readily than elusive threatened wildlife. However, reservoir hosts can be hard to recognise [5] and, in some cases, there may be no external reservoir [8].

Population crashes and local extinctions of endangered African wild dogs (*Lycaon pictus*) have been linked to canine pathogens such as rabies virus [9], [10], canine distemper virus [11], [12] and the bacterial pathogen *Ehrlichia canis* [13]. Canine parvovirus and the protozoan pathogen *Neospora caninum* have been associated with mortality of wild dog pups [14], [15], a demographic impact which limited the growth of grey wolf (*Canis lupus*) populations [16]. Other pathogens found in wild dogs, such as canine coronavirus [17], might also reduce survival and hence influence population viability. All of these pathogens can infect domestic dogs as well as wild dogs, and domestic dogs are often assumed to function as reservoirs of infection [4], [18].

The dynamics of infectious diseases are also likely to be strongly influenced by wild dogs' behaviour and ecology. Wild dogs occur at low population densities, living in highly cohesive territorial social groups (packs) [19], [20], [21], [22]. Simple epidemiological models predict that the high contact rates which occur within social groups will elevate the prevalence of directly transmitted infections [23], a prediction which is broadly supported by empirical data [24]. Hence, exposure might be especially high in large wild dog packs once a pathogen is introduced. However, encounters between packs are rare due to low population density and territorial behaviour, so there may be few opportunities for pathogen transmission between packs [25]. These behavioural effects on contact patterns suggest that intraspecific pathogen transmission is likely to be frequency-dependent rather than density-dependent [26].

Here, we assess the importance of within- and between-species transmission of pathogens in African wild dogs coexisting with domestic dogs. We describe patterns of exposure to six canine pathogens with diverse transmission mechanisms: rabies virus and canine distemper virus, which are transmitted by direct contact [27], [28]; parvovirus and coronavirus, which are transmitted through faeces [29], [30]; *Ehrlichia canis*, which is transmitted by a tick vector [31]; and *Neospora caninum*, which may be transmitted by consumption of infected prey or by contact with infected faeces, as well as across the placenta from mother to foetus [32]. For each pathogen, we compare individual wild dogs' risk of exposure with behavioural measures of their opportunities for transmission from (i) within their own pack; (ii) other wild dog packs; and (iii) domestic dogs.

## Methods

### Ethics statement

Animals were captured and handled in collaboration with the Kenya Wildlife Service, with permission from the Kenyan Ministry of Science and Technology (permit MOEST 13/001/32C 47) as well as private and community landholders, according to guidelines of the IUCN/SSC Canid Specialist Group, and following a protocol approved by the Animal Care and Use Committee of the University of California, Davis (protocol 10813), and the Ethics Committee of the Zoological Society of London (project BPE/0510).

### Study area and study populations

The study was conducted in 2001–9 in northern Kenya, in Laikipia District (37° 2′ E, 0° 6′ N, 1800m ASL), and parts of neighbouring Samburu and Isiolo Districts. The core study area is 4,500 km^2^ of semi-arid bush land (mean annual rainfall 590mm), used for subsistence pastoralism, commercial ranching, and tourism.

Wild dogs disappeared from the study area in the 1980s, but recolonised naturally in the late 1990s [33]. Wild dog density increased through the course of the study, rising from 0.009 adults and yearlings/km^2^ in 2001 to 0.034 adults and yearlings/km^2^ in 2009 [33]. Mean wild dog pack size in the area was 9.1 adults and yearlings (range 3–21) and mean litter size (at three months of age) was 7.3 (range 2–14 [33]). Annual mortality of radio-collared adults and yearling wild dogs was 29%, with disease associated with 40% of known-cause deaths [33]. Wild dog packs occupied overlapping territories averaging 278 km^2^ (range 60–718km^2^), with territory size unrelated to pack size [20].

The study area comprises two main land use types. Privately-owned commercial ranches form a contiguous block in the south-west of the study area, with the remainder being community lands occupied by Samburu and Masai pastoralists. Human and livestock densities are substantially higher on community lands than on commercial ranches, with densities of wild dog prey correspondingly lower [34]. Wild dog pack territories can be clearly identified as falling on one or the other land use type [20], [33], but population characteristics and ranging patterns are similar in the two land uses [20], [33]. Local people keep domestic dogs in both land use types, for security at bomas (livestock corrals where most people and domestic dogs reside) and to accompany grazing herds. Average domestic dog densities are substantially higher on community lands (3.39 domestic dogs/km^2^) than on commercial ranches (0.21 domestic dogs/km^2^) [25].

### Wild dogs

In 2001–9, 90 wild dogs in 19 packs were immobilized to fit radio-collars for monitoring purposes. Capture methods are detailed in ref [33]; most wild dogs were darted from a stationary vehicle at distances of 10–20m. All captured wild dogs were immobilized by intramuscular administration of medetomidine (Domitor, Pfizer Animal Health; approximately 26 µg/kg) and ketamine (approximately 2.6 mg/kg), and reversed with atipamezole (Antisedan, Pfizer Animal Health; approximately 130 µg/kg, also intramuscular). Of these 90 animals, 16 were immobilized twice and three were immobilized three times to replace expired or damaged radio-collars.

While wild dogs were immobilized, blood samples were collected from the jugular vein, into 10ml evacuated serum separator tubes (Vacutainers, Becton-Dickinson, Oxford, UK). Blood samples were allowed to clot before being centrifuged; serum was then removed and aliquots were stored at −20°C.

Wild dogs fitted with radio-collars were monitored using aerial and ground-based telemetry. Aerial telemetry was conducted approximately weekly, usually between 0700–0830h (during wild dogs' morning hunting period), and provided locations with an accuracy of around 200m. Packs including radio-collared animals were visited regularly on the ground to monitor pack size, membership, and reproductive state.

Of 90 wild dogs captured, 33 were of known age, having been previously identified (from their unique pelage patterns) as pups (<1 year) with birth dates known from regular pack monitoring with a precision of a few days. The ages of a further 12 wild dogs could be confidently estimated, having been first identified as pups or yearlings (≥1 year, <2 years, recognisable during handling based on body dimensions and tooth wear), with birth dates estimated as 12 months prior to their pack's next recorded breeding attempt (since wild dogs breed approximately annually). The ages of the remaining 45 wild dogs, first identified as adults (≥2 years), were estimated using a combination of tooth wear, pelage characteristics, reproductive state and social status.

None of the wild dogs in this study had been vaccinated against any pathogen.

### Domestic dogs

During 2001–9, blood samples were collected from 184 domestic dogs which owners reported had no history of vaccination against any pathogen. Of these domestic dogs, 121 were sampled at 75 bomas throughout the study area, and 63 were sampled at 14 locations on community lands within the study area where free rabies vaccination was being provided annually. Domestic dogs were manually restrained and blood was collected from the cephalic vein into 10ml evacuated tubes (Vacutainers, Becton-Dickinson, Oxford, UK). Blood samples were allowed to clot before being centrifuged; serum was then removed and aliquots were stored at −20°C.

Data on the densities and distribution of domestic dogs were available from a survey of 639 bomas, described in ref [25]. Data on domestic dog movement patterns were available from GPS collars fitted to 15 domestic dogs in 2004–5 (details in ref [25]).

### Serological analyses

For all serological analyses, threshold titres interpreted as evidence of prior pathogen exposure were selected to optimise test sensitivity and consistency with other studies. Additionally, the effects of choosing different threshold titres were explored in statistical analyses (see below).

Serum samples were screened for antibodies to rabies virus, using a rapid fluorescent focus inhibition test (RFFIT) at the Centers for Disease Control and Prevention, Atlanta, GA [35]. In primary statistical analyses, RFFIT titres >0.05 IU/ml were interpreted as likely evidence of prior exposure to rabies virus [36]. Although established infection with rabies virus has been viewed as fatal in canids, antibodies have been found in unvaccinated free-ranging African wild dogs [9], domestic dogs [37], [38], black-backed jackals [39] and Ethiopian wolves [40], as well as in spotted hyenas [41]. Serological analysis was therefore expected to provide useful information on exposure to rabies virus.

Serum samples were screened for antibodies to canine distemper virus, using a serum neutralisation (SN) test and the Onderstepoort virus strain, at the Animal Health Diagnostic Center at Cornell University [42]. Samples were also screened for antibodies to canine coronavirus using a SN test at the same laboratory, using virus strain S378/6. For both SN tests, in primary statistical analyses animals with antibodies detectable at dilutions ≥1∶8 were considered likely to have been exposed to the respective viruses.

Serum samples were screened for antibodies to canine parvovirus, using a haemagglutination inhibition (HAI) test, again at the Animal Health Diagnostic Center at Cornell University [43]. In primary statistical analyses, antibodies detectable at titres ≥1∶20 were considered likely to indicate prior exposure.

Serum samples were screened for antibodies to *Ehrlichia canis* and *Neospora caninum* using indirect fluorescent antibody (IFA) tests at the School of Veterinary Medicine, University of California, Davis [44], [45]. In primary statistical analyses, antibodies detectable at titres ≥1∶40 were considered likely to indicate prior exposure.

### Contact among wild dogs

We investigated three potential measures of contact among wild dogs. First, we used pack size (measured as numbers of adults and yearlings, and numbers of pups, at the time of sampling) as an index of individual wild dogs' day-to-day probability of contact with conspecifics. Second, we used the date of sampling (measured in days since 1 Jan 2001) as a proxy to estimate contact probability, since wild dog population size, density, and home range overlap all increased over time [20], [33]. Third, we estimated wild dogs' risk of contact with other packs in the 12 months prior to sampling, using aerial telemetry data. Full details of the estimation method are given in ref [25] but, in brief, we used nine years of aerial radio-telemetry data to estimate the average frequency of contact between neighbouring wild dog packs per active period, and then multiplied this frequency by the number of packs with home ranges overlapping or adjoining that of the sampled wild dog in the 12 months before sampling. This risk of contacting other packs was only estimated for wild dogs with known movement patterns over the previous 12 months, that is, animals which throughout this period either were radio-collared themselves, or were known to have been members of a pack with at least one radio-collared member..

### Contact between wild dogs and domestic dogs

Since domestic dog densities are higher on community lands than on commercial ranches [25], the land use type inhabited by each wild dog gave one measure of risk of contact with domestic dogs.

In addition to this simple binary measure, we derived a continuous measure of domestic dog contact risk for each wild dog sampled. Full details of this method are given in ref [25] but, in brief, we estimated the density of domestic dogs in areas used by wild dogs, by (i) calculating the proximity of each wild dog pack aerial telemetry location to nearby bomas; (ii) using a function relating domestic dog density to distance from the boma – based on GPS-collar tracking of domestic dogs – to estimate domestic dog density at each pack location; and (iii) averaging across all aerial telemetry locations recorded in the 12 months prior to sampling (mean number of locations per wild dog pack  = 35.6, SD = 13.6). This estimate of domestic dog contact risk was only calculated for wild dogs with known movement patterns over the previous 12 months.

### Statistical analyses

Primary analyses of serological data considered the proportions of wild dogs showing evidence of exposure to the particular pathogens, as indicated by having antibodies detectable at dilutions greater than the thresholds indicated above. These analyses were conducted using mixed logistic regression models, including pack identity as a random effect, using the *lmer* procedure in *R* (http://lme4.r-forge.r-project.org/). Results are reported as odds ratios (OR) and related 95% confidence intervals (CI) associated with doubling the values of the explanatory variables (risk factors).

Continuous variables which were not normally distributed were log-transformed. To avoid problems associated with zero values, a number equivalent to half the lowest non-zero value was added to all values before taking the (natural) logarithms. Where animals had been sampled on more than one occasion, data from the most recent date were used in statistical analyses, to maximise available data on the animals' history prior to sampling. In addition, data on repeated sampling of the same animals were used to investigate seroconversion (by comparing data from the first and last sampling event).

Preliminary analyses used mixed logistic regression models (including pack identity as a random effect) to investigate, separately, the potential effects on pathogen exposure of (i) individual characteristics (sex, age in months); (ii) risk of contacting other wild dogs (measured as pack size, sampling date {a proxy for overall population density}, and mean risk of contacting another wild dog pack in the previous 12 months); and (iii) risk of domestic dog contact (measured as land use type {commercial ranch/community land}, and as domestic dog density experienced in the previous 12 months). For *Neospora*, we also investigated possible vertical transmission of infection by including the mother's serological status in analyses (where known). Variables with *p*≥0.15 in these preliminary analyses were entered into multivariable analyses, with potential explanatory variables eliminated sequentially until only statistically significant effects remained. Statistical analyses were restricted to animals with complete data on all hypothesised covariates; this gave sample sizes of 57 animals for the viral pathogens and *Ehrlichia*, and 38 animals for *Neospora*. For maximum precision, descriptive analyses include all sampled individuals.

Exploratory analyses were also conducted to investigate the effects of using alternative threshold values for considering animals exposed (using mixed logistic regression), and, alternatively, using the (log-transformed) antibody titres as continuous outcome variables (using generalised linear mixed models {GLMM}, including pack identity as a random effect, using the *lme* procedure in *R* [46]). These exploratory analyses gave results which were qualitatively the same as the primary analyses, and so are not presented here. Generalised linear mixed models, including pack identity as a random effect, were likewise used to explore correlations between the two measures of domestic dog contact risk, and among the three measures of wild dog contact risk.

## Results

### Correlations among measures of contact risk

The three measures of intraspecific contact among wild dogs were correlated with one another. Pack size increased over time, whether measured as the number of adults and yearlings (GLMM, effect of days since 1 Jan 2001, p = 0.002), or as the number of pups (GLMM, effect of days since 1 Jan 2001, p = 0.006). The estimated risk of contacting another wild dog pack likewise increased over time (GLMM, effect of days since 1 Jan 2001, p<0.001). However, after adjusting for the effects of time, the estimated risk of contacting another wild dog pack was not significantly related to pack size, whether measured as the number of adults and yearlings (GLMM including days since 1 Jan 2001, effect of adult and yearling number p = 0.16) or as the number of pups (p = 0.11).

**Figure 1:**
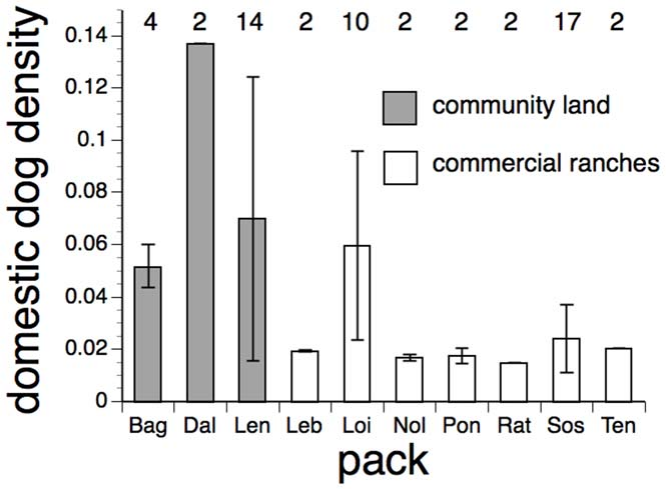
Density of domestic dogs experienced by 57 African wild dogs, in 10 packs living mainly on either community lands or commercial ranches. Data show the mean (and SD) estimated density of domestic dogs at points where wild dogs were located by aerial radio-telemetry in the 12 months prior to sampling for pathogen exposure. Figures along the top of the graphs indicate the numbers of wild dogs sampled from each pack (over periods of 1–7 years).

There was likewise evidence of correlation between the two measures of wild dogs' risk of contact with domestic dogs. The domestic dog density experienced in the 12 months prior to sampling was higher for 20 wild dogs living mainly on community lands, than for 37 living mainly on commercial ranches (Figure 1; GLMM of log-transformed domestic dog contact risk, effect of land use type, p = 0.008).

### Exposure to canine pathogens

**Figure 2:**
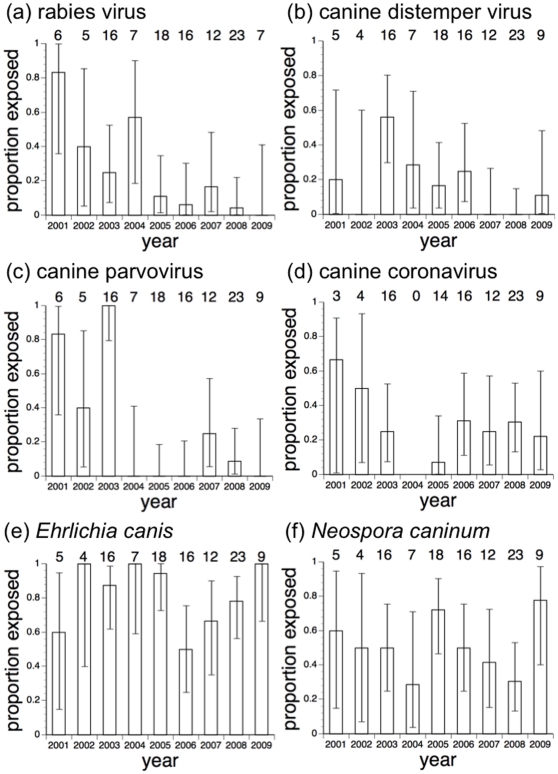
Inter-annual variation in the proportions of African wild dogs showing evidence of exposure to (a) rabies virus; (b) canine distemper virus; (c) canine parvovirus; (d) canine coronavirus; (e) *Ehrlichia canis*; and (f) *Neospora caninum*. Error bars indicate exact binomial confidence intervals. Figures along the tops of the graphs indicate the numbers of samples screened in each year; the sums of these figures exceed the denominators in Table 1 because they include samples from wild dogs immobilized multiple times.

**Figure 3:**
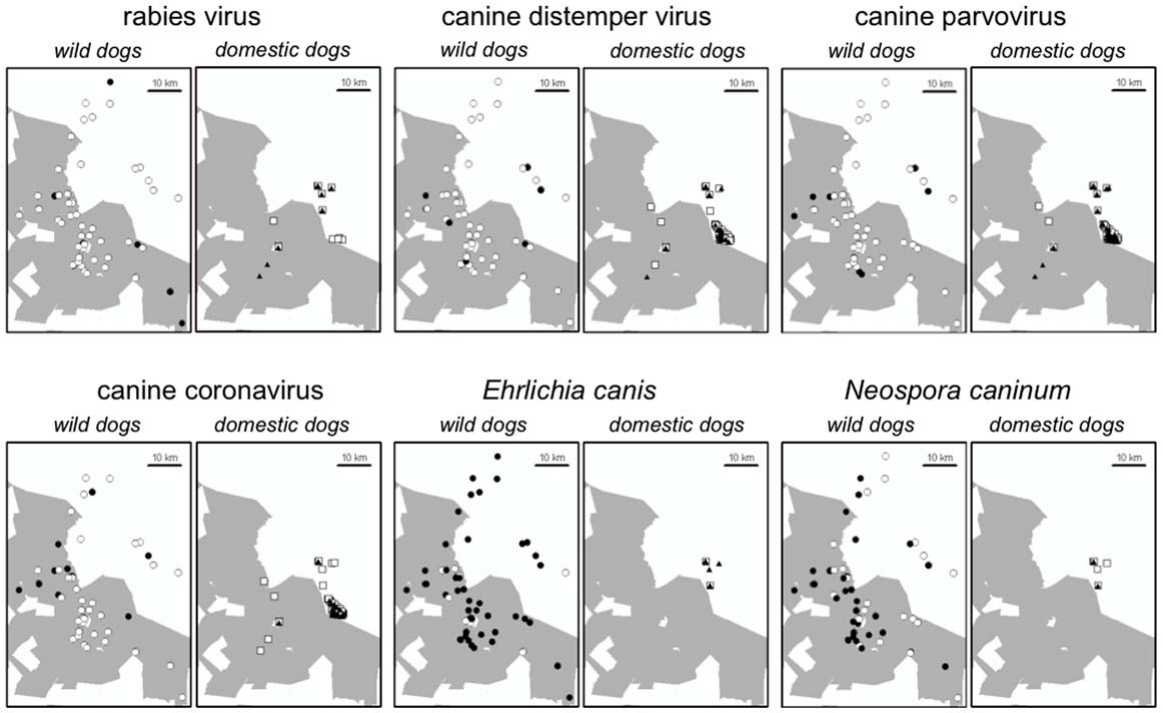
Spatial distribution of pathogen exposure among wild dogs and domestic dogs. Maps show, for each pathogen, the sampling locations for animals with (filled symbols) and without (open symbols) evidence of exposure. Shading indicates commercial ranch land. Note that multiple animals were sampled at some locations.

Table 1 shows the proportions of wild dogs and domestic dogs with evidence of exposure to the six canine pathogens on the most recent sampling date. Populations of both host species were exposed to all of the pathogens investigated. Table 2 shows how such evidence of exposure varied among wild dogs sampled more than once. For all pathogens except rabies virus, seroconversion from negative to positive was observed, providing evidence of likely exposure to the pathogens in the course of the study. (We also recorded sporadic deaths from confirmed rabies in the course of the study [47], [48], suggesting that this pathogen was also circulating at the time of the study). For all pathogens except *Neospora*, seroconversion from positive to negative was observed, providing evidence of titres fading over time (though not necessarily indicating loss of immunity). Figure 2 shows temporal variation in the proportions of wild dogs with evidence of pathogen exposure, and Figure 3 shows spatial variation in exposure among both wild dogs and domestic dogs sampled.

**Table 1:** Proportions of wild dogs and domestic dogs with serological evidence of exposure to six canine pathogens on their most recent sampling date.

Pathogen	Wild dog		Domestic dog	
	*+ve/tested*	*seroprevalence*	*+ve/tested*	*seroprevalence*
rabies virus	13/88	15%	24/82	29%
canine distemper virus	14/88	16%	88/184	48%
canine parvovirus	22/89	25%	117/183	64%
canine coronavirus	21/83	25%	28/184	15%
*Ehrlichia canis*	70/88	80%	56/65	86%
*Neospora caninum*	45/87	52%	12/65	18%

None of these animals had any history of vaccination to any pathogen.

**Table 2:** Changes in the serological status of wild dogs which were sampled twice, on dates 2.5–38 months apart.

	unchanged		seroconverted		
Pathogen	*negative*	*positive*	*to positive*	*to negative*	total
rabies virus	11	2	0	4	17
canine distemper virus	12	2	1	4	19
canine parvovirus	13	0	4	2	19
canine coronavirus	4	4	2	3	13
*Ehrlichia canis*	0	13	5	1	19
*Neospora caninum*	8	8	3	0	19

Animals which seroconverted were considered negative when first sampled, but positive subsequently, or *vice versa*. None of these animals was vaccinated against any pathogen.

There was a non-significant trend suggesting that the proportion of wild dogs exposed to rabies virus may have been higher among wild dogs with greater opportunities for contact with domestic dogs (Table 3). No other covariates improved the fit of this model.

**Table 3:** Results of multivariable logistic regression models describing predictors of wild dog exposure to six canine pathogens.

Pathogen	Risk factor	OR (95% CI)	P
rabies virus	domestic dog contact	1.95 (0.91–4.18)	0.088
canine distemper virus	year 2003 *vs* other years	32.0 (2.8–360.1)	<0.001
canine parvovirus	adult pack size	0.23 (0.08–0.67)	0.007
	domestic dog contact	8.66 (1.59–219.02)	0.020
canine coronavirus	age (in months)	1.07 (1.02–1.13)	0.009
	days since 1 Jan 2001	0.997 (0.995–1.000)	0.036
*Ehrlichia canis*	adult pack size	0.77 (0.63–0.95)	0.016
	domestic dog contact	3.43 (1.01–11.70)	0.049
*Neospora caninum*	adult pack size	0.27(0.09–0.81)	0.019
	domestic dog contact	8.53 (3.40–67.7)	0.015

All models also include pack identity as a random effect. For risk factors measured as continuous variables, odds ratios describe the effects of a doubling in value.

The proportion of wild dogs exposed to canine distemper virus declined over time (mixed logistic regression model, effect of days since 1 Jan 2001, OR = 0.999, CI = 0.998–1.000, p = 0.036). However, the temporal pattern suggested that prevalence peaked in 2003 (Figure 2(b)), and a binary variable distinguishing 2003 from all other years described the data better than the continuous variable “days since 1 Jan 2001” (mixed logistic regression model, 2003 *vs* other years, OR = 32.0, CI = 2.8–360.1, p<0.001 (Table 3); mixed logistic regression model including both binary “year” variable and continuous time variable, effect of binary “year” variable p = 0.061, effect of continuous time variable p = 0.68).

The proportion of wild dogs with evidence of exposure to parvovirus was greater in small packs, with greater opportunities for contact with domestic dogs (Table 3).

The proportion of wild dogs with evidence of exposure to canine coronavirus was higher in older animals, and also declined over time (Table 3).

The proportion of wild dogs with evidence of exposure to *Ehrlichia* was greater in small packs with greater opportunities for contact with domestic dogs (Table 3).

The proportion of wild dogs with evidence of exposure to *Neospora* was greater in small packs with greater opportunities for contact with domestic dogs (Table 3). Mothers' exposure to *Neospora* significantly increased the probability of exposure among their offspring in preliminary analyses (including only this variable and pack identity as a random effect; OR = 32.0, CI = 1.8–583.9, p = 0.019), but this effect became non-significant when other variables were added (p = 0.14 when included alongside adult pack size and domestic dog contact).

## Discussion

Our findings are consistent with the hypothesis that domestic dogs transmit canine pathogens to wild dogs. Wild dogs with greater opportunities for contact with domestic dogs were at greater risk of exposure to canine parvovirus, *Ehrlichia*, *Neospora*, and possibly rabies virus, four pathogens with diverse transmission mechanisms. In contrast, there were no links between domestic dog contact risk and wild dogs' probability of exposure to canine distemper virus or canine coronavirus, although these viruses did occur in the domestic dog population, and have transmission mechanisms similar to those of the pathogens which were associated with domestic dogs.

We found a clearer link between pathogen exposure and domestic dog density within this population than in a parallel study which compared exposure across populations, using protected area status as a proxy for domestic dog contact [49]. This difference probably reflects the more fine-grained data on domestic dog contact which were available in this study; we note that our continuous measure of domestic dog contact (based on particular packs' ranging behaviour) was also a better predictor of pathogen exposure than was our binary land use variable, even though these two explanatory variables were correlated with one another.

The lack of any association between wild dogs' exposure to canine distemper virus and their contact with domestic dogs is consistent with recent evidence suggesting that wildlife may play an important role in maintaining this pathogen, both in this study area and elsewhere [36], [49], [50]. The lack of any link between wild dogs' exposure to coronavirus and their contact with domestic dogs is more surprising, since a comparison across sites did detect such an association [49]. The proportion of domestic dogs exposed to coronavirus was lower than the corresponding proportion of wild dogs (Table 1), and there was also evidence to suggest that this pathogen may have been spatially clustered within the domestic dog population (Figure 3). These patterns suggest that some wild dogs may have been contacting uninfected domestic dogs, perhaps explaining the lack of association between domestic dog contact and wild dog exposure to this pathogen.

Our findings revealed no evidence that wild dogs' contact with other members of the same species increased their exposure to canine pathogens. Contrary to expectation, exposure to parvovirus, *Ehrlichia* and *Neospora* were associated with small pack size (rather than large pack size as would be expected if high intra-pack contact increased exposure). Although such negative associations between social group size and pathogen exposure are unusual [24], they have been observed in other host-pathogen systems [51]. One potential explanation for these negative associations is that parvovirus, *Ehrlichia* and *Neospora* might increase wild dog (especially pup) mortality [13], [14], [15], [16] and hence themselves suppress pack size. Since pack size is unrelated to home range size in this population [20], variation in home range size is unlikely to explain these negative associations. Rising wild dog density over time was likewise not linked to increasing pathogen exposure. Contrary to expectation, exposure to coronavirus declined significantly over time, and similar patterns (albeit non-significant) were observed for all of the viral pathogens (Figure 2). We speculate that these patterns might reflect gradual fading-out of infections following recolonisation of the study area from community lands [33] where average rates of domestic dog contact, and hence pathogen exposure, may have been higher. The behavioural measure of inter-pack contact was not significantly associated with exposure to any of the pathogens.

Our finding that wild dogs' exposure to several pathogens was elevated by contact with domestic dogs is consistent with, though not sufficient to confirm, the hypothesis that domestic dogs function as reservoir hosts for these pathogens. Empirical data on both contact rates [25] and pathogen exposure [36] suggest that, in this study area, domestic dogs do act as a reservoir for rabies virus: infection appears to persist in the domestic dog population, but not in the wild dog population [36], probably because domestic dogs occur at high densities with high intraspecific contact rates, whereas wild dogs live at low population densities with few opportunities for transmission between packs [25]. In contrast, the other pathogens found to be associated with domestic dog contact – parvovirus, *Ehrlichia* and *Neospora* – might in principle be maintained in low-density host populations, like those of wild dogs, without the involvement of another primary host species [52]. This is partly because their transmission does not require direct contact between hosts, being able to survive in, respectively, the environment, a tick vector, and ungulate prey [52] and, in the case of *Neospora*, also being vertically transmitted [32]. It is possible that domestic dog populations function as reservoirs for these pathogens but, alternatively, domestic dog contact may simply elevate the prevalence of these infections among wild dogs, without being necessary for infection to persist. The latter appears especially likely for *Neospora*, which occurs at higher prevalence in wild dogs than in domestic dogs (Table 1), with high prevalence perhaps being maintained by vertical transmission. We note that exposure to all of the pathogens considered here has been recorded among wild dogs inhabiting large protected areas remote from domestic dogs [9], [14], [17], [49], [53]; hence the absence of domestic dogs does not guarantee total protection from any of these pathogens.

Since the pathogens associated with domestic dog contact have all been linked to wild dog mortality, the viability of wild dog populations might be undermined by contact with domestic dogs. However, exposure to these pathogens – and by extension contact with domestic dogs – is not necessarily harmful in all cases. Indeed, the fact that we detected evidence of exposure to these pathogens in apparently healthy wild dogs, in a growing population, shows that exposure is not invariably fatal, and did not cause population decline at the level of domestic dog contact observed here. Exposure to these pathogens may maintain some level of immunity in the population, helping to prevent large outbreaks from occurring or mitigating their lethality, and may also help to maintain selection pressure for disease resistance. The balance between these positive and negative effects is likely to vary between pathogens, suggesting that a single management strategy (e.g., domestic dog removal) might not be appropriate for all pathogens. However, substantial increases in contact between wild dogs and domestic dogs – as might occur through land use change [25] – would be expected to increase pathogen exposure for wild dogs and could undermine population viability.

Our findings help to identify the most appropriate pathogen-specific control measures. Among the pathogens we studied, rabies virus has the clearest record of causing mortality in wild dogs [9], [10], [54], [55], [56] and is thus the most likely to require management intervention. The findings presented here, combined with those from other studies [25], [36], [57] suggest that, in principal, wild dogs might (where necessary) be protected from rabies by vaccinating either domestic dogs or wild dogs themselves. Both of these approaches have strengths and weaknesses: domestic dog vaccination can fail to protect wildlife if conducted over too small an area [2], but has the added advantage of protecting human health [58], whereas wild dog vaccination directly targets the host of conservation concern but has proven controversial in the past [59]. Deciding which form of management is most appropriate therefore requires a careful analysis of costs and benefits. Canine distemper virus is less likely than rabies virus to require management, since it has less of a history of causing wild dog mortality (but see [11], [12]). However, should this virus seriously threaten a wild dog population, vaccination of domestic dogs might have no conservation benefit since there is growing evidence to suggest that this pathogen is not always maintained in domestic dog populations [36], [49], [50]. Protecting wild dogs from canine distemper would therefore need to target wild dogs themselves. For the other pathogens studied here, both disease dynamics and impacts on wild dog populations and are less certain; hence, deciding on the most appropriate form of management (if any) remains problematic.

This study is one of a very small number of empirical attempts to quantify rates of contact between wild and domestic mammals [60], [61], [62]. To our knowledge, this is the first such study to successfully link individual variation in hosts' opportunities for interspecific contact to variation in pathogen exposure. Identifying such links required detailed data on the behaviour of both host species, highlighting one practical challenge of recognising reservoirs of pathogens affecting wildlife [5], even when one of the hosts is a domestic species. Still greater challenges would be involved when both hosts are wildlife (but see [63]). Nevertheless, further studies of this kind are needed to address the growing number of wild species threatened by infectious disease [64].

## References

[1] Tompkins DM, Sainsbury AW, Nettleton P, Buxton D, Gurnell J (2002). Parapoxvirus causes a deleterious disease in red squirrels associated with UK population declines.. Proceedings of the Royal Society of London Series B-Biological Sciences.

[2] Randall DA, Marino J, Haydon DT, Sillero-Zubiri C, Knobel DL (2006). An integrated disease management strategy for the control of rabies in Ethiopian wolves.. Biological Conservation.

[3] Thorne ET, Williams ES (1988). Disease and endangered species: The black-footed ferret as a recent example.. Conservation Biology.

[4] Cleaveland S, Hess GR, Dobson AP, Laurenson MK, McCallum HI, Hudson PJ, Rizzoli A, Grenfell B, Heesterbeck H (2002). The role of pathogens in biological conservation.. The Ecology of Wildlife Diseases.

[5] Haydon DT, Cleaveland S, Taylor LH, Laurenson MK (2002). Identifying reservoirs of infection: A conceptual and practical challenge.. Emerging Infectious Diseases.

[6] Hudson P, Greenman J (1998). Competition mediated by parasites: biological and theoretical progress.. Trends in Ecology and Evolution.

[7] Woodroffe R (1999). Managing disease risks to wild mammals.. Animal Conservation.

[8] Hawkins CE, Baars C, Hesterman H, Hocking GJ, Jones ME (2006). Emerging disease and population decline of an island endemic, the Tasmanian devil *Sarcophilus harrisi*.. Biological Conservation.

[9] Gascoyne SC, King AA, Laurenson MK, Borner M, Schildger B (1993). Aspects of rabies infection and control in the conservation of the African wild dog (*Lycaon pictus*) in the Serengeti region, Tanzania.. Onderstepoort Journal of Veterinary Research.

[10] Hofmeyr M, Bingham J, Lane EP, Ide A, Nel L (2000). Rabies in African wild dogs (*Lycaon pictus*) in the Madikwe Game Reserve, South Africa.. Veterinary Record.

[11] Alexander KA, Kat PW, Munson LA, Kalake A, Appel MJG (1996). Canine distemper-related mortality among wild dogs (*Lycaon pictus*) in Chobe National Park, Botswana.. Journal of Zoo & Wildlife Medicine.

[12] Goller KV, Fyumagwa RD, Nikolin V, East ML, Kilewo M (2010). Fatal canine distemper infection in a pack of African wild dogs in the Serengeti ecosystem, Tanzania.. Veterinary Microbiology.

[13] Stevenson-Hamilton J (1939). The health of wild animals.. Journal of the South African Veterinary Medical Association.

[14] Creel S, Creel NM, Munson L, Sanderlin D, Appel MJG (1997). Serosurvey for selected viral diseases and demography of African wild dogs in Tanzania.. Journal of Wildlife Diseases.

[15] Woodroffe R, Ginsberg JR, Macdonald DW (1997). The African wild dog: Status survey and conservation action plan..

[16] Mech LD, Goyal SM, Paul WJ, Newton WE (2008). Demographic effects of canine parvovirus on a free-ranging wolf population over 30 years.. Journal of Wildlife Diseases.

[17] van Heerden J, Mills MGL, Van Vuuren MJ, Kelly PJ, Dreyer MJ (1995). An investigation into the health status and diseases of wild dogs (*Lycaon pictus*) in the Kruger National Park.. Journal of the South African Veterinary Medical Association.

[18] Alexander K, Appel M (1994). African wild dogs (*Lycaon pictus*) endangered by a canine distemper epizootic among domestic dogs near the Masai Mara National Reserve, Kenya.. Journal of Wildlife Diseases.

[19] Mills MGL, Gorman ML (1997). Factors affecting the density and distribution of wild dogs in the Kruger National Park.. Conservation Biology.

[20] Woodroffe R (2011). Ranging behaviour of African wild dog packs in a human-dominated landscape.. Journal of Zoology.

[21] Fuller TK, Kat PW, Bulger JB, Maddock AH, Ginsberg JR, McCullough DR, Barrett H (1992). Population dynamics of African wild dogs.. Wildlife 2001: Populations.

[22] Creel S, Creel NM (2002). The African wild dog: behavior, ecology and conservation..

[23] Anderson RM, May RM (1979). Population biology of infectious diseases, I. Nature.

[24] Coté IM, Poulin R (1995). Parasitism and group size in social animals – a meta-analysis.. Behavioral Ecology.

[25] Woodroffe R, Donnelly CA (2011). Risk of contact between endangered African wild dogs *Lycaon pictus* and domestic dogs: opportunities for pathogen transmission.. Journal of Applied Ecology.

[26] Begon M, Bennett M, Bowers RG, French NP, Hazel SM (2002). A clarification of transmission terms in host-microparasite models: numbers, densities and areas.. Epidemiology and Infection.

[27] Fekadu M, Baer GM (1991). Canine rabies.. The Natural History of Rabies.

[28] Appel MJ, Appel MJ (1987). Canine distemper virus.. Virus infections of carnivores.

[29] Appel MJ, Parrish CR, Appel MJ (1987). Canine parvovirus type 2.. Virus infections of carnivores.

[30] Appel MJ, Appel MJ (1987). Canine coronavirus.. Virus infections of carnivores.

[31] Stich RW, Schaefer JJ, Bremer WG, Needham GR, Jittapalapong S (2008). Host surveys, ixodid tick biology and transmission scenarios as related to the tick-borne pathogen, *Ehrlichia canis*.. Veterinary Parasitology.

[32] Dubey JP, Schares G, Ortega-Mora LM (2007). Epidemiology and control of neosporosis and *Neospora caninum*.. Clinical Microbiology Reviews.

[33] Woodroffe R (2011). Demography of a recovering African wild dog (*Lycaon pictus*) population.. Journal of Mammalogy.

[34] Woodroffe R, Lindsey PA, Romañach SS, ole Ranah SMK (2007). African wild dogs (*Lycaon pictus*) can subsist on small prey: implications for conservation.. Journal of Mammalogy.

[35] Smith JS, Yager PA, Baer GM (1973). Rapid reproducible test for determining rabies neutralizing antibody.. Bulletin of the World Health Organization.

[36] Prager KC, Mazet JK, Dubovi EJ, Frank LG, Munson L

[37] Cleaveland S, Barrat J, Barrat MJ, Selve M, Kaare M (1999). A rabies serosurvey of domestic dogs in rural Tanzania: results of a rapid fluorescent focus inhibition test (RFFIT) and a liquid-phase blocking ELISA used in parallel.. Epidemiology and Infection.

[38] Cleaveland S, Barrat J, Barrat MJ, Selve M, Kaare M (1999). A rabies serosurvey of domestic dogs in rural Tanzania: results of a rapid fluorescent focus inhibition test (RFFIT) and a liquid-phase blocking ELISA used in parallel.. Epidemiology and Infection.

[39] Alexander KA, Kat PW, Wayne RK, Fuller TK (1994). Serologic survey of selected canine pathogens among free-ranging jackals in Kenya.. Journal of Wildlife Diseases.

[40] Sillero-Zubiri C, King AA, Macdonald DW (1996). Rabies and mortality in Ethiopian wolves (*Canis simensis*).. Journal of Wildlife Diseases.

[41] East ML, Hofer H, Cox JH, Wulle U, Wiik H (2001). Regular exposure to rabies virus and lack of symptomatic disease in Serengeti spotted hyenas.. Proceedings of the National Academy of Sciences, USA.

[42] Appel MJG, Robson DS (1973). A microneutralization test for canine distemper virus.. American Journal of Veterinary Research.

[43] Carmichael LE, Joubert JC, Pollock RV (1980). Hemagglutination by canine parvovirus: serologic studies and diagnostic applications.. American Journal of Veterinary Research.

[44] Ristic M, Weisiger RM, Huxsoll DL, Nyindo MBA, Hildebra PK (1972). Serological diagnosis of tropical canine pancytopenia by indirect immunofluorescence.. Infection and Immunity.

[45] Holmberg TA, Vernau W, Melli AC, Conrad PA (2006). *Neospora caninum* associated with septic peritonitis in an adult dog.. Veterinary Clinical Pathology.

[46] Laird NM, Ware JH (1982). Random-effects models for longitudinal data.. Biometrics.

[47] Woodroffe R, Chapman K (2006). African wild dogs and African people: conservation through coexistence..

[48] Woodroffe R (2009). African wild dogs and African people: conservation through coexistence..

[49] Prager KC, Mazet JAK, Munson L, Dubovi EJ, Szykman Gunther M (in review) The effect of protected areas on pathogen exposure in endangered African wild dog (*Lycaon pictus*) populations..

[50] Craft ME, Hawthorne PL, Packer C, Dobson AP (2008). Dynamics of a multihost pathogen in a carnivore community.. Journal of Animal Ecology.

[51] Woodroffe R, Donnelly CA, Wei G, Cox DR, Bourne FJ (2009). Social group size affects *Mycobacterium bovis* infection in European badgers (*Meles meles*).. Journal of Animal Ecology.

[52] Lloyd-Smith JO, Cross PC, Briggs CJ, Daugherty M, Getz WM (2005). Should we expect population thresholds for wildlife disease?. Trends in Ecology and Evolution.

[53] Prager KC (2011). An investigation of infectious disease dynamics in African carnivores: identifying reservoirs and risk Factors and Investigating control strategies [PhD]..

[54] Kat PW, Alexander KA, Smith JS, Munson L (1995). Rabies and African wild dogs in Kenya.. Proceedings of the Royal Society of London B.

[55] Hofmeyr M, Hofmeyr D, Nel L, Bingham J (2004). A second outbreak of rabies in African wild dogs (*Lycaon pictus*) in Madikwe Game Reserve, South Africa, demonstrating the efficacy of vaccination against natural rabies challenge.. Animal Conservation.

[56] Scheepers JL, Venzke KAE (1995). Attempts to reintroduce African wild dogs *Lycaon pictus* into Etosha National Park, Namibia.. South African Journal of Wildlife Research.

[57] Prager KC, Woodroffe R, Cameron A, Haydon DT (2011). Vaccination strategies to conserve the endangered African wild dog (*Lycaon pictus*).. Biological Conservation.

[58] Knobel DL, Cleaveland S, Coleman PG, Fevre EM, Meltzer MI (2005). Re-evaluating the burden of rabies in Africa and Asia.. Bulletin of the World Health Organization.

[59] Woodroffe R (2001). Assessing the risks of intervention: immobilization, radio-collaring and vaccination of African wild dogs.. Oryx.

[60] Ruttimann S, Giacometti M, McElligott AG (2008). Effect of domestic sheep on chamois activity, distribution and abundance on sub-alpine pastures.. European Journal of Wildlife Research.

[61] Richomme C, Gauthier D, Fromont E (2006). Contact rates and exposure to inter-species disease transmission in mountain ungulates.. Epidemiology and Infection.

[62] Courtenay O, Quinnell R, Chalmers WSK (2001). Contact rates between wild and domestic canids: no evidence of parvovirus or canine distemper virus in crab-eating foxes.. Veterinary Microbiology.

[63] Formenty P, Boesch C, Wyers M, Steiner C, Donati F (1999). Ebola virus outbreak among wild chimpanzees living in a rain forest of Cote d'Ivoire.. Journal of Infectious Diseases.

[64] Leendertz FH, Pauli G, Maetz-Rensing K, Boardman W, Nunn C (2006). Pathogens as drivers of population declines: The importance of systematic monitoring in great apes and other threatened mammals.. Biological Conservation.

